# Social support buffers the negative influence of perceived injustice on pain interference in people living with HIV and chronic pain

**DOI:** 10.1097/PR9.0000000000000710

**Published:** 2019-03-14

**Authors:** Terence M. Penn, Zina Trost, Romy Parker, William P. Wagner, Michael A. Owens, Cesar E. Gonzalez, Dyan M. White, Jessica S. Merlin, Burel R. Goodin

**Affiliations:** a Department of Psychology, University of Alabama at Birmingham, Birmingham, AL, USA; b Department of Anesthesia and Perioperative Medicine, University of Cape Town, Cape Town, South Africa; c Divisions of General Internal Medicine and Infectious Diseases, Department of Medicine, University of Pittsburgh, Pittsburgh, PA, USA

**Keywords:** HIV, Chronic pain, Perceived injustice, Social support, Pain interference

## Abstract

**Introduction:**

A growing literature attests to the overwhelming prevalence of disabling chronic pain among people living with HIV (PLWH), yet very little is known about psychosocial contributors to poor chronic pain outcomes in this population. Pain-related perception of injustice may promote pain interference by hindering engagement in daily activities among individuals with chronic pain. Social support has been shown to buffer the negative impact of harmful beliefs on well-being and facilitate adjustment to chronic pain.

**Objective:**

This cross-sectional study tested the buffering hypothesis of social support to determine whether increasing levels of social support mitigate the negative influence of perceived injustice on pain interference.

**Methods:**

A total of 60 PLWH with chronic pain completed measures of perceived injustice, social support, pain severity, and interference, as well as depressive symptoms.

**Results:**

In a regression-based model adjusted for age, sex, depressive symptoms, and pain severity, results indicated that social support significantly moderated (ie, buffered) the association between perceived injustice and pain interference (*P* = 0.028). Specifically, it was found that perceived injustice was significantly associated with greater pain interference among PLWH with low levels of social support (*P* = 0.047), but not those with intermediate (*P* = 0.422) or high levels of social support (*P* = 0.381).

**Conclusion:**

Pain-related injustice perception reflects harmful beliefs regarding severity of loss consequent to chronic pain development, a sense of unfairness, and irreparability of loss. Access to a social support network may provide an adaptive means of mitigating the negative effects of perceived injustice.

## 1. Introduction

Chronic pain is becoming increasingly recognized as a common and debilitating health comorbidity among people living with HIV (PLWH). A recent review of the literature indicated that chronic pain may affect over half of all PLWH throughout their lifetimes, with increasing prevalence as these individuals age.^35^ People living with HIV also endure high-impact chronic pain that interferes with general physical and mental health, as well as HIV-specific clinical outcomes. For example, chronic pain in PLWH is associated with greater odds of functional impairment,^29^ increased health care service utilization,^23^ suboptimal retention in HIV care,^31^ and failure to achieve virologic suppression.^27^ Despite increasing research efforts focused on the prevalence and consequences of chronic pain in PLWH, still very little is known about the role of psychosocial processes in the development and maintenance of chronic pain in this population.^32^

One psychosocial factor that may contribute to chronic pain outcomes in PLWH is perception of pain-related injustice, which has been conceptualized as a set of cognitive appraisals reflecting the severity and irreparability of pain-related loss, externalized blame, and unfairness.^38,43^ Emerging research findings suggest that perceived injustice consequent to chronic pain development might contribute to more severe pain interference beyond what can be attributed to pain severity.^44^ Pain interference refers to the self-reported consequences of pain on relevant aspects of one's life including engagement in emotional, physical, cognitive, social, and recreational activities.^1^ A high degree of perceived injustice is also consistently associated with greater pain report,^48^ depressive and post-traumatic symptoms,^39,45^ as well as poorer treatment outcomes, such as following multidisciplinary pain rehabilitation.^40,45^ No study to date has examined perceived injustice among PLWH in relation to pain interference or other pain-relevant clinical outcomes.

When examining the negative impact of perceived injustice on chronic pain outcomes in PLWH, it is likely important to consider resiliency factors that may lessen the deleterious effects of perceived injustice. Social support is a resiliency factor that has been shown to be associated with better adjustment to chronic pain by promoting adaptive coping skills.^16,41^ Moreover, the stress-buffering hypothesis of social support posits that access to social support can eliminate or weaken the negative effects of a perceived stressor on health and quality of life.^6,13^ Social support refers to the perception and actuality that one is cared for, has assistance available from other people, and is part of a supportive social network. Five general categories of social support have been conceptualized, including emotional (eg, nurturance), tangible (eg, financial assistance), informational (eg, advice), social network support (eg, sense of belonging), and esteem (eg, validation).^8^ Taken together, it may be that social support provided by the friends, family, and significant others of PLWH with chronic pain confers benefit by mitigating the negative influence of perceived injustice on pain interference. Alternatively, PLWH with chronic pain who have very little social support may experience a significantly greater impact of their perceived injustice pain interference.

**Figure 1.**
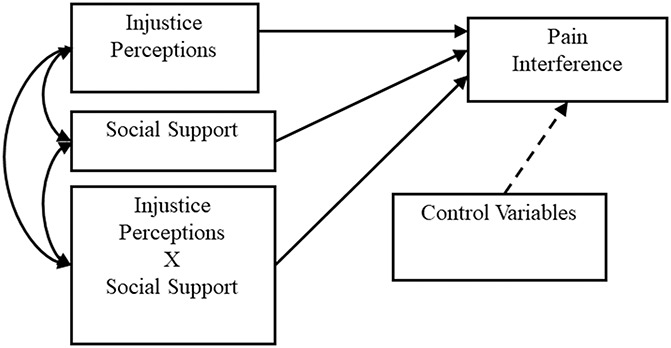
Flow diagram depicting participant matriculation through the study.

This cross-sectional study sought to examine associations among perceived injustice, social support, and pain interference in a sample of PLWH with chronic pain. Figure 1 displays the putative conceptual model for these associations. Two primary hypotheses were tested. (1) Greater perceived injustice is associated with greater pain interference across the entire study sample. (2) Social support will moderate (ie, buffer) the association between perceived injustice and pain interference, such that greater perceived injustice will be associated with greater pain interference, but only for those with low social support.

## 2. Methods

### 2.1. Participants and procedures

**Figure 2.**
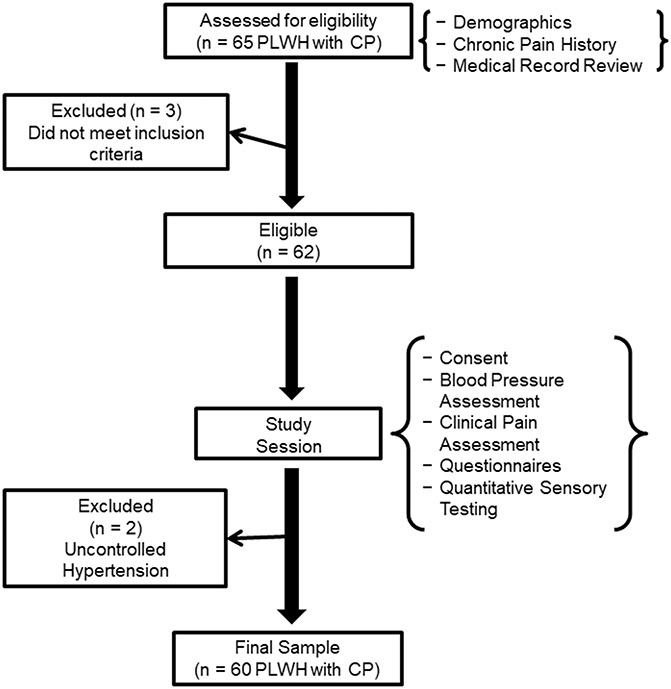
Moderation model representing the interaction between perception of pain-related injustice and social support in relation to pain interference. CP, chronic pain.

The current study is part of a larger completed project that sought to examine risk and resiliency factors related to the experience of chronic pain in PLWH (Comprehensive HIV and Pain Study). Figure 2 presents a flow diagram depicting participant matriculation through the study. A total of 65 PLWH with chronic pain were recruited from a large, urban HIV clinic in the Southeast United States that provides comprehensive medical, behavioral, and social services to approximately 3,500 adults (≥18 years) with HIV. The final sample size was 60 PLWH with chronic pain. Chronic pain status was assessed by asking participants 2 questions: (1) “How long have you had chronic pain?” and (2) “How long has chronic pain been an ongoing problem for you over the past 6 months?” Chronic pain was defined as bodily pain that had persisted for at least 3 consecutive months and that was present on at least half the days in the past 6 months.^47^ Those interested in study participation completed a telephone screening to determine initial eligibility. Potential participants were excluded if their self-reported health history was positive for other conditions that could potentially confound pain interference or otherwise impede their ability to reliably complete the study measures. These conditions included evidence of uncontrolled hypertension (ie, resting blood pressure >150/95), history of cardiac events/disease, history of stroke or seizure, and history of cancer. Sociodemographic information was collected from all participants and included age, sex (man, women, and transgender), and racial background (non-Hispanic black and non-Hispanic white), as well as poverty status. Annual household income adjusted for number of occupants was used to determine poverty status according to 2017 guidelines put forth by the US Department of Health and Human Services.^10^ All study procedures were approved by the local Institutional Review Board and performed in accordance with guidelines for the ethical conduct of research.

### 2.2. Medical record review

Medical record review was completed to ascertain the rates of psychiatric diagnoses among participants, as well as to determine whether participants were actively being prescribed medications that could affect their pain, such as analgesics or antidepressants. Medical records were also used to obtain most recent CD4^+^ T-cell count, a measure of immune status, and most recent viral load value. Those PLWH with ≥200 copies of virus/mL were considered to have a detectable viral load; all others were considered virologically suppressed (ie, without detectable viral loads). Finally, medical record review was used to confirm participants' self-reported health history provided during telephone screening. Those PLWH whose medical records corroborated their self-reported health history, and who met study inclusion criteria, were deemed eligible for ongoing participation. Eligible participants were subsequently scheduled for a single study session at the Biobehavioral Pain Research Laboratory (PI: B.R.G.) to complete the measures listed below. Written informed consent was obtained from each participant at the beginning of the study session, and they were compensated for their involvement.

### 2.3. Measures

#### 2.3.1. Pain interference

The Brief Pain Inventory-Short Form (BPI-SF) is a self-administered questionnaire used to measure pain interference and pain severity over the past 24 hours.^5,24^ The pain interference dimension of the BPI-SF comprises 7 items measuring the degree to which pain interferes with functioning in the following domains: general activity, mood, walking ability, normal work, relations with other persons, sleep, and enjoyment of life. Level of interference is measured using a rating scale from 0 (no interference) to 10 (complete interference), and the mean score denotes the total level of pain interference. The BPI-SF has been widely used and validated as a measure of pain interference in daily functioning.^46^ In this study, the BPI-SF was used specifically to address pain interference, but not pain severity. This was performed to minimize common-method variance between these 2 dimensions when including both pain interference and pain severity in the same data analytic model. The pain interference dimension of the BPI-SF used in this study demonstrated excellent internal consistency (Cronbach's α = 0.92).

#### 2.3.2. Pain severity

At the beginning of the study, all participants self-reported on the severity of their chronic pain by responding to the following single item, “Please rate the severity of any bodily pain you have experienced during the past week.” Response options included 0 (None), 1 (very mild), 2 (mild), 3 (moderate), 4 (severe), 5 (very severe). Responses to this item were included in data analytic models as an index of chronic pain severity.

#### 2.3.3. Perceived injustice

The Injustice Experience Questionnaire (IEQ) was used to assess perception of pain-related injustice in this sample of PLWH.^43^ Participants rated the frequency with which they experienced each of 12 thoughts/feelings when reflecting on their chronic pain condition. Items are rated on a scale of 0 (never) to 4 (all of the time). Injustice Experience Questionnaire items broadly reflect the associated factors of “severity/irreparability of loss” and “blame/unfairness.” Representative severity/irreparability items include “Most people don't understand how severe my condition is, and “My life will never be the same.” Blame/unfairness items include “I am suffering because of someone else's negligence, and “It all seems so unfair.” The IEQ has demonstrated strong psychometric properties, including sensitivity to change, among individuals with persistent musculoskeletal pain.^43,48^ Cronbach's alpha for IEQ in the current study was 0.88, indicating good internal consistency.

#### 2.3.4. Social support

Perceived social support among PLWH was addressed using the Multidimensional Scale of Perceived Social Support (MSPSS).^50^ This scale consisted of 12 items that measure the extent of social support received from 3 specific sources: friends, family, and significant others. Types of social support assessed by the MSPSS included emotional (eg, “I get the emotional help and support I need from my family”), tangible (eg, “There is a special person who is around when I am in need”), informational (eg, “My family is willing to help me make decisions”), social network support (eg, “I can count on my friends when things go wrong”), and esteem (eg, “I have a special person who is a real source of comfort to me”). Each item was scored on a scale ranging from 1 (very strongly disagree) to 7 (very strongly agree). Summation of the 12 item scores provided a possible total score ranging from 12 to 84 for overall social support, with higher scores corresponding to higher levels of social support. The MSPSS measure used in this study demonstrated excellent internal consistent according to Cronbach's alpha = 0.95.

#### 2.3.5. Depression

Depressive symptoms were assessed using the Center for Epidemiological Studies-Depression Scale (CES-D).^36^ This 20-item measure assesses the frequency of experiencing depressive symptoms over the past week 0 (never or rarely) to 3 (most of the time/all the time). Symptoms of depression measured by the CES-D include negative mood, guilt/worthlessness, helplessness/hopelessness, psychomotor retardation, loss of appetite, and sleep disturbance. This measure has been shown to be reliable and valid in general populations, including when used in HIV and chronic pain populations.^18,34^ Responses are summed (range 0–60), with higher scores indicating greater severity of depression. The CES-D measure used in the current study had good internal consistency (Cronbach's α = 0.88).

### 2.4. Data reduction and analysis

All data were analyzed using SPSS, version 24 (IBM, Chicago, IL). Alpha level for determining significance was set at *P* < 0.05. All participants provided complete demographic data (eg, sex, age, ethnicity/race, and household income); however, a small portion of missing data existed for the self-report questionnaires (<5% for any one questionnaire). Data were deemed to be missing at random; and therefore, a simple data imputation method was completed using the macro for hot deck imputation.^33^ This data imputation method is well validated and accepted in the statistical community^2^ and resulted in complete study data for each of the 60 participants. To test the first hypothesis, Pearson's correlations were used to evaluate the zero-order association between perceived injustice and pain interference, as well as associations among all other continuously measured variables. Group differences across categorical variables (eg, sex and ethnicity/race) were examined using 1-way analysis of variance. To test the second hypothesis, the PROCESS macrocreated and described by Hayes^20^ was used to examine whether the relationship between perceived injustice and pain interference was significantly moderated by social support. The Johnson-Neyman technique was then applied to probe the interaction between perceived injustice and social support using bootstrapped confidence intervals with 5,000 resamples.^20^ This technique tests the significance of the conditional association between perceived injustice and pain interference within the observed range of values of the moderator (social support) until the value of the moderator is identified, for which the conditional association is just statistically significant at a set level (α = 0.05). Values of the moderator for which the conditional association is significant constitute the region of significance.

## 3. Results

### 3.1. Participant characteristics

Descriptive characteristics for the 60 study participants are presented in Table 1. The mean age was 47.6 years (SD = 8.6). The sample of PLWH comprised 54% men, 39% women, and 7% transgender women, whereas 92% were non-Hispanic black and the remaining 8% were non-Hispanic white. The mean CD4^+^ count was 664 (SD = 335), 8% had a detectable viral load (>200 copies/mL), and 98% were prescribed antiretroviral therapy. The most frequently reported location of chronic pain was low back (44%), hips/knees (30%), neck (9%), widespread (3+ sites) (9%), shoulder/arm/hand (4%), and feet (4%). Approximately 3/4 of the sample (73.3%) reported their pain severity over the past week as either severe or very severe. Sixty-one percent of the sample was prescribed analgesic medication for pain, and the most common prescription was nonsteroidal anti-inflammatory drugs (33%). Seventy-five percent of the sample had a psychiatric diagnosis listed in their medical record, with the most common diagnosis being depression (36%). Approximately 46% of PLWH were actively prescribed an antidepressant medication, with the most common prescriptions being selective serotonin reuptake inhibitors and serotonin and norepinephrine reuptake inhibitors (38%).

**Table 1:**
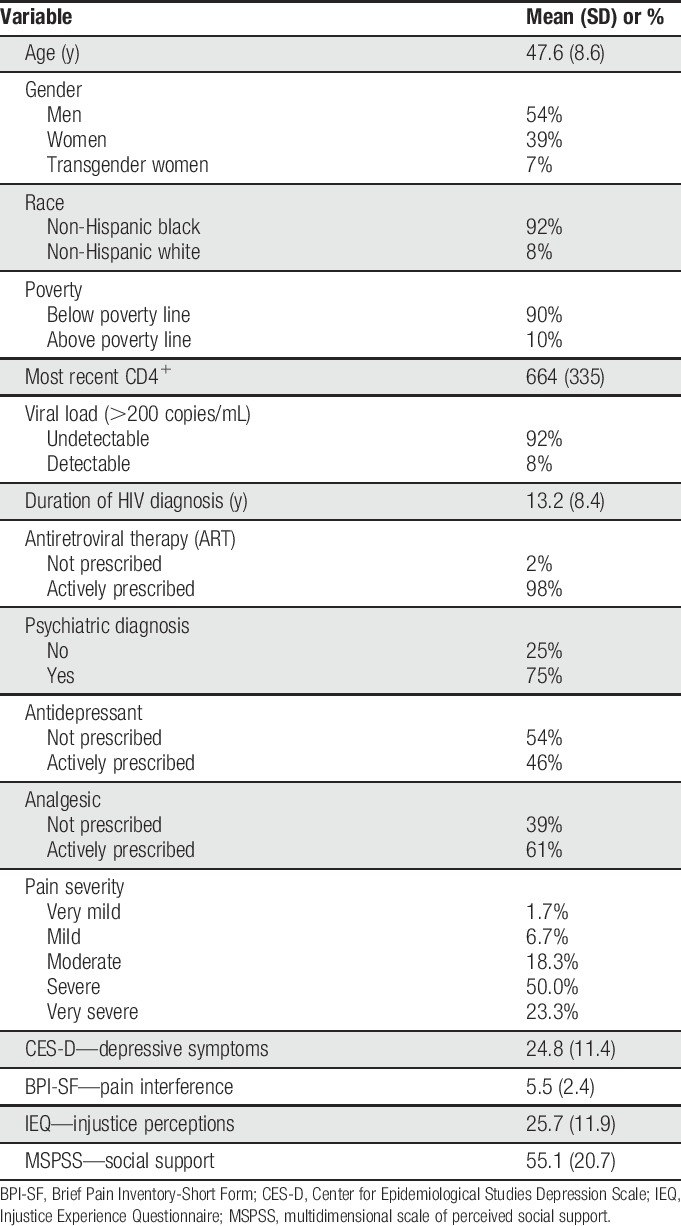
Participant characteristics.

### 3.2. Group differences and zero-order correlations

Women reported significantly greater pain interference compared with men (*P* = 0.046), but not compared with transgender women (*P* = 0.103). The pain interference reported by non-Hispanic black and white PLWH did not significantly differ (*P* = 0.426). Pain interference did not significantly differ according to whether participants were actively being prescribed an analgesic (*P* = 0.968) or antidepressant (*P* = 0.477). Similarly, the pain interference reported by PLWH with a psychiatric diagnosis listed in their medical chart was not significantly different from those without a psychiatric diagnosis (*P* = 0.389). Finally, pain interference was comparable between PLWH with a detectable viral load and those who were suppressed (*P* = 0.998).

Zero-order Pearson correlations among continuously measured variables are presented in Table 2. Greater perceived injustice was significantly correlated with more pain interference (*P* = 0.039) and less perceived social support (*P* = 0.023). Depressive symptoms were significantly correlated with greater perceived injustice (*P* < 0.001), less social support (*P* = 0.009), greater pain severity (*P* = 0.032), and greater pain interference (*P* = 0.001). Greater pain severity was also significantly correlated with greater pain interference (*P* = 0.011). Of note, a partial correlation analysis controlling for pain severity and depressive symptoms revealed that perceived injustice was no longer significantly associated with pain interference (*r*_partial_ = 0.087, *P* = 0.515).

**Table 2:**
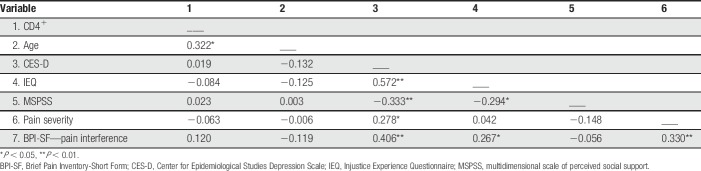
Pearson's correlations among continuously measured variables.

### 3.3. Moderation

**Figure 3.**
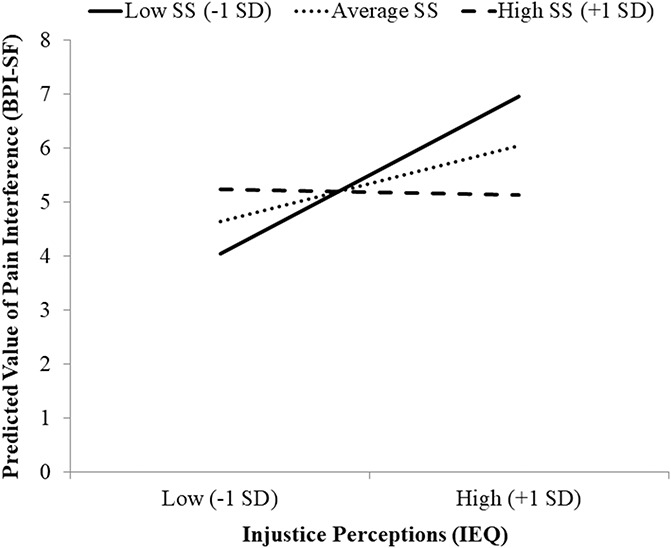
Plot of simple slopes depicting associations between perception of pain-related injustice and pain interference at low, average, and high levels of social support (SS).

The following covariates were included in the moderation analysis: age, dummy-coded gender, pain severity, and depressive symptoms. The moderation analysis was completed to determine whether the strength of the association between perceived injustice and pain interference differed according to level of social support reported by the participants. The overall moderation model adjusted for covariates (Table 3) accounted for a significant 37% of the variance in pain interference (*R*^2^ = 0.371, *P* = 0.002). There was a significant interaction between perceived injustice and social support in relation to pain interference (*R*^2^Δ = 0.063, *P* = 0.028), suggesting that social support indeed moderated (ie, buffered) the association between perceived injustice and pain interference (*P* = 0.028). Specifically, as demonstrated in Figure 3, it was found that perceived injustice was significantly associated with greater pain interference among PLWH who reported low levels of social support (*P* = 0.047), but not those who reported average (*P* = 0.422) or high levels of social support (*P* = 0.381). Finally, the results of the Johnson-Neyman technique (Table 4) show that, as the amount of social support increases, the association between perceived injustice and pain interference weakened and became nonsignificant.

**Table 3:**
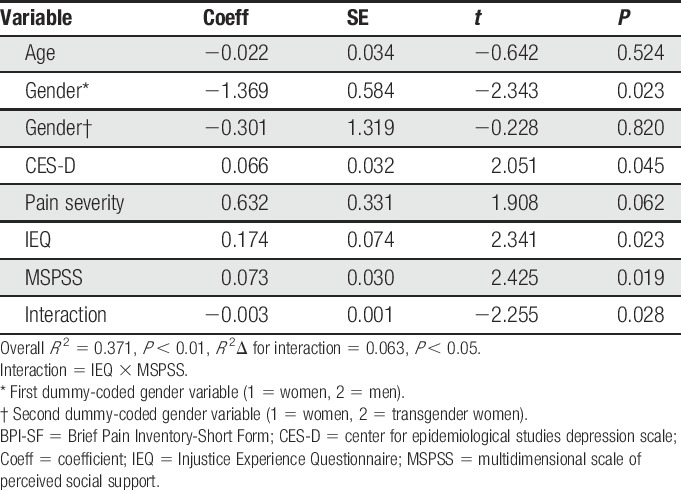
Moderation analysis testing whether social support (MSPSS) and injustice perceptions (IEQ) are interactively related to pain interference (BPI-SF) adjusting for covariates.

**Table 4:**
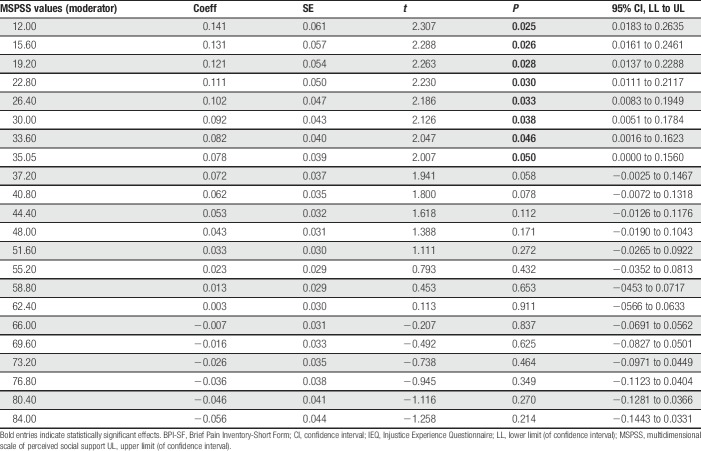
Johnson-Neyman technique demonstrating the moderated effect of injustice perceptions (IEQ) on pain interference (BPI-SF) across observed values for social support (MSPSS).

## 4. Discussion

In support of our first hypothesis, perception of pain-related injustice was found to be significantly correlated (zero-order) with pain interference across the entire sample of PLWH with chronic pain. However, in the partial correlation controlling for pain severity and depressive symptoms, perception of pain-related injustice was no longer associated with pain interference. This suggests that pain severity and depressive symptoms may be important factors to consider when examining the impact of perceived pain-related injustice on pain interference in future research. Findings further revealed that the amount of social support perceived from friends, family members, and significant others significantly buffered the observed association between perceived injustice and pain interference, which supports our second hypothesis. In particular, perceived injustice was significantly associated with greater pain interference among individuals with low levels of social support, but not those with average or high levels of social support. Our findings remained significant even after statistically controlling for age, sex differences, depressive symptoms, and pain severity. Taken together, these findings lend support to our study hypotheses and suggest that a high-quality social support network may provide an adaptive means of mitigating the negative effects of perceived injustice on chronic pain outcomes. Our interpretation of these findings is consistent with extant literature describing the stress-buffering hypothesis of social support.

### 4.1. Perceived injustice in people living with HIV with chronic pain

This is the first study of its kind to demonstrate a significant association between perception of pain-related injustice and the extent to which pain interferes with daily functioning in PLWH with chronic pain. In this study, the mean level of perceived injustice reported on the IEQ measure (25.7, SD = 11.9) was higher than what has been reported in previous studies that consisted of non-HIV adult samples with chronic low-back pain (21.9, SD = 13.1),^49^ whiplash injury (16.2, SD = 8.3),^4^ fibromyalgia (21.6, SD = 9.7), and rheumatoid arthritis (14.4, SD = 9.8).^17^ One possible reason for elevated perception of pain-related injustice in our sample may be related to social stigma experienced by PLWH with HIV. The existing literature is replete with studies demonstrating the negative physical and mental health effects of HIV stigma.^14,15^ However, PLWH are also susceptible to experiencing intersectional stigmas, which can arise at the juncture of multiple stigmatized identities and/or health conditions.^9,37^ To illustrate, recent research completed by our study team has found that PLWH with chronic pain experience a high degree of stigma related to their chronic pain, as well as their HIV status.^19^

Although some PLWH with chronic pain have clear pathophysiological causes of pain (eg, avascular necrosis), for many, there is no clear association between pain and identifiable pathology. Medically unexplained pain is often termed “nonspecific” and prone to the stigmatizing reactions of others.^12^ This is because observers tend to react with uncertainty and confusion to individuals whose pain in not clearly medically understood.^12^ Previous research has shown that laypersons and providers alike are less inclined to help, feel less sympathy, dislike patients more, suspect deception, and attribute lower pain severity to individuals whose pain does not have an objective basis in tissue pathology.^11^ Because of these stigmatizing responses from others, PLWH with chronic pain may feel that their pain is being devalued and discredited. As a result, PLWH with chronic pain who experience a high degree of stigma may be particularly susceptible to engaging the cognitive appraisals (ie, severity and irreparability of pain-related loss, externalized blame, and unfairness) that conceptually characterize perception of pain-related injustice. Whether experiences of chronic pain stigma drive perceived injustice in PLWH remains hypothetical and is a question worthy of future research.

### 4.2. Beneficial effects of social support

Individuals with chronic pain who report high levels of social support experience less distress and less severe pain, with higher levels of support associated with better adjustment.^26^ People living with HIV with chronic pain may seek social support as one strategy for pain self-management.^28^ For example, in a recent study of men living with HIV, intensive experience sampling methods were used to understand the relationships between moment-to-moment experiences of social support and pain.^7^ Findings revealed that perceived social support and feelings of acceptance were associated with less pain within individuals over time.

The buffering hypothesis is one explanation addressing how social support protects (or “buffers”) people from the deleterious effects of stressful life events such as living with chronic pain.^6^ Specifically, social support promotes adaptive appraisal and coping, which in turn mitigates the impact of stressful life events on health.^22^ By promoting adaptive appraisals and coping, exposure to high levels of social support may offset the deleterious effects of perceived injustice on pain interference, as observed in the current study of PLWH with chronic pain.

### 4.3. Clinical implications

These findings have implications for the assessment and management of chronic pain among PLWH. Pain and its interference with the physical and mental functioning of PLWH should be widely assessed by health care providers. Results of this study suggest that it would likely be important for health care providers to prioritize identifying and providing support to those PLWH with chronic pain who report the lowest amounts of social support derived from friends, family, and significant others. By incorporating an empathic and collaborative approach to pain assessment and management, PLWH are likely to feel validated and cared for by their health care provider, which in turn should promote a strong therapeutic alliance between provider and patient.^25,42^ Health care providers may then provide HIV-related social support, in addition to helping PLWH feel supported when addressing their concerns about chronic pain.^21^ Feeling supported by their health care provider may serve as an effective substitute for the low levels of social support derived from friends, family, and significant others. As a result of feeling supported by their health care provider, PLWH may be more likely to take an active role in the day-to-day management of their pain.^3^

Until recently, there existed no evidence-based behavioral pain management interventions for populations affected by HIV. To address this need, a 12-session pain self-management intervention, “Skills TO Manage Pain,” was developed that combines individual coping skills development with group sessions to bolster peer support around the experience of living with chronic pain and HIV (clinicaltrials.gov NCT02824562).^30^ The feasibility of incorporating Skills TO Manage Pain has been demonstrated,^28^ and currently a large scale study is underway to test the efficacy and effectiveness of this behavioral pain intervention tailored to PLWH. Our findings highlight the importance of integrating social support into pain management to optimize intervention effects. Specific content addressing how to effectively seek out social support could be integrated into individual- and group-based pain management formats. If feasible, health care providers should consider including a family member, friend, or significant other in sessions to facilitate communication about pain and how to provide appropriate support.

### 4.4. Study limitations

This study has several limitations that should be considered when interpreting the results, and that future research may expand upon. First, we did not assess the types of social support perceived by PLWH with chronic pain in this study, but future research could determine whether the type of social support (eg, tangible, emotional, informational, and esteem) differentially modifies the effect of perceived injustice on pain-related functional and psychosocial outcomes. Second, the cross-sectional design of this study precludes our ability to form conclusions about whether perception of pain-related injustice indeed predicts the chronification of pain that interferes with daily living. Alternatively, it is possible that PLWH with greater pain interference in this study were more likely to perceive their chronic pain as unjust and unfair. Future longitudinal research could address whether perceived injustice at baseline predicts subsequent pain interference with physical, cognitive, and emotional functioning over time, as well as the ways whereby procurement of social support mitigates the effects of perceived injustice in PLWH with chronic pain. Finally, it is not clear from this study whether the ability of social support to buffer the negative impact of perceived injustice on pain is specific to PLWH or might be found in other non-HIV populations with chronic pain. Future work should test whether our results can be replicated among larger, more diverse chronic pain populations with and without HIV to determine the generalizability of these findings. Despite these limitations, results of this study contribute to a stronger understanding of psychosocial risk (eg, perceived injustice) and resiliency (eg, social support) factors in relation to chronic pain outcomes among PLWH.

## 5. Conclusion

Perception of pain-related injustice reflects maladaptive beliefs regarding severity of loss consequent to chronic pain development, a sense of unfairness, and irreparability of loss. Findings support the contribution of perceived injustice to pain interference in daily living among PLWH with chronic pain above and beyond depressive symptoms and pain severity. Importantly, a high-quality social support network may provide an adaptive means of mitigating the negative effects of perceived injustice in this population. The current findings support the need for evidence-based behavioral pain management intervention that combines individual coping skills development with group sessions to bolster peer support around the experience of living with chronic pain and HIV.

## Disclosures

The authors have no conflict of interest to declare.
